# Exercise addiction: is it linked to eating disorders? A cross-sectional study in a sample of Tunisian athlete students

**DOI:** 10.1192/j.eurpsy.2024.614

**Published:** 2024-08-27

**Authors:** N. Smaoui, O. Bouattour, R. Feki, I. Gassara, M. Maalej, N. Charfi, J. Ben Thabet, M. Maalej, S. Omri, L. Zouari

**Affiliations:** ^1^psychiatry C department, Hedi chaker university hospital, sfax, Tunisia

## Abstract

**Introduction:**

Eating disorders and sports addiction are becoming increasingly common among athletes.It’s important to be aware of these disorders in order to improve their overall prevention.

**Objectives:**

The aim of our study was to determine the links between exercise addiction (EA) and eating disorders in Tunisian students at the Institute of Physical Education and to examine the factors associated with these disorders.

**Methods:**

An anonymous self-administered questionnaire was distributed to students in the Sfax and Gafsa sports sections during March 2023. The Exercise Addiction Inventory (EAI) was used to study exercise addiction. It is a scale whose purpose is to separate individuals into 3 groups: those at risk of exercise addiction (score ≥ 24), those non-addicts with symptoms (score 13 to 23) and those non-addicts without symptoms (score 0 to 12).

Eating disorders were assessed using the SCOFF-F questionnaire ( Sick, Control, One stone, Fat, Food), with a score of 2 or more indicating possible eating disorders.

**Results:**

We collected 240 participants. The mean SCOFF-F and EAI scores were 1.7±1.3 and 16.6 ± 4.1 respectively. Among the participants, 52.9% of students were at risk of developing eating disorders and 2.5% of students were at risk of exercise addiction. In our study, 82.5% of students took part in regular physical activity in a gym. The main reasons for going to the gym were muscle strengthening (57.9%) and preparation for a sporting competition (37%).

Among students exercising outside the institute, the mean SCOFF score was significantly higher for those doing so to prepare for a sports competition (p=0.001), for professional obligations (p=0.005) or for weight loss (p=0.001). Participants at risk of exercise addiction had a higher mean SCOFF score, but the difference was not significant (p=0.051).

**Conclusions:**

Our study shows that eating disorders were widespread among Tunisian athlete students, and were higher among students at risk of exercise addiction.

**Disclosure of Interest:**

None Declared

